# Influence of image reconstruction kernel on computed tomography-based finite element analysis in the clinical opportunistic screening of osteoporosis—A preliminary result

**DOI:** 10.3389/fendo.2023.1076990

**Published:** 2023-03-01

**Authors:** Chenyu Jiang, Dan Jin, Ming Ni, Yan Zhang, Huishu Yuan

**Affiliations:** Department of Radiology, Peking University Third Hospital, Beijing, China

**Keywords:** osteoporosis, finite element, vertebral fracture, opportunistic screening, computed tomography

## Abstract

**Purpose:**

This study aimed to evaluate the difference in vertebral mechanical properties estimated by finite element analysis (FEA) with different computed tomography (CT) reconstruction kernels and evaluate their accuracy in the screening and classification of osteoporosis.

**Methods:**

There were 31 patients enrolled retrospectively from the quantitative CT database of our hospital, uniformly covering the range from osteoporosis to normal. All subjects’ CT raw data were reconstructed both with a smooth standard convolution kernel (B40f) and a sharpening bone convolution kernel (B70f), and FEA was performed on L1 of each subject based on two reconstructed images to obtain vertebral estimated strength and stiffness. The trabecular volumetric bone mineral density (vBMD) of the same vertebral body was also measured. FEA measurements between two kernels and their accuracy for osteoporosis screening were compared.

**Results:**

The vertebral stiffness and strength measured in FEA-B40f were significantly lower compared with those of FEA-B70f (12.0%, *p* = 0.000 and 10.7%, *p* = 0.000, respectively). The correlation coefficient between FEA-B70F and vBMD was slightly higher than that of FEA-B40F in both vertebral strength and stiffness (strength: *r* ^2^-B40f = 0.21, *p* = 0.009 vs. *r* ^2^-B70f = 0.27, *p* = 0.003; stiffness: *r* ^2^-B40f = 0.37, *p* = 0.002 vs. *r* ^2^-B70f = 0.45, *p*=0.000). The receiver operator characteristic curve showed little difference in the classification of osteoporosis between FEA-B40f and FEA-B70f.

**Conclusion:**

Two kernels both seemed to be applicable to the opportunistic screening of osteoporosis by CT-FEA despite variance in FE-estimated bone strength and bone stiffness. A protocol for CT acquisition and FEA is still required to guarantee the reproducibility of clinical use.

## 1 Introduction

Osteoporosis is a skeletal disorder characterized by compromised bone strength and an increased risk of fracture (1). Although areal bone mineral density (aBMD) assessed by dual-energy X-ray absorptiometry (DXA) is a standard clinical protocol for estimating fracture risk, it has been criticized for low accuracy and underutilization. Nearly half of the fragility fractures occur in individuals with normal aBMD, and 21% to 50% of patients with fragility fractures had a femoral neck BMD in the range of osteoporosis (2–4). On the other hand, although DXA was the most meaningful examination for osteoporosis, more than 60% of patients had never undergone DXA before or after fragility fractures (5). Conversely, accessibility to computed tomography (CT) scans are markedly better (6), and approximately 54.5% of those CT scans are performed at relevant osteoporotic fracture sites (7). In 2015, the International Society of Clinical Densitometry identified the priority of quantitative computed tomography (QCT) for its use in fracture prediction (8), as it simultaneously allows DXA-equivalent femoral aBMD, volumetric BMD (vBMD), and finite element analysis (FEA).

The FE method has been used to simulate the mechanical behavior of bone with increasing fidelity for decades, which has shown great reliability to assess bone strength and fracture risk (8–10). Previous studies confirmed the superiority of FEA to DXA-aBMD (11) and even QCT-vBMD (12) in both prevalent and incident vertebral fracture prediction (13–16). Aside from fracture prediction, FEA has a unique value in the opportunistic screening of osteoporosis (7), drug efficacy (17), and postoperative evaluation of internal fixation (18).

However, material property mapping for the finite elements entails conversion from CT attenuation Hounsfield units (HU) to Young’s modulus through empirical equations, which thus introduces variability in fracture risk prediction from CT acquisition protocols. It is well established that CT acquisition, including tube current (mAs), voltage (kVp), reconstruction algorithms, and scanner type, will affect the grayscale value measured in HU (19, 20). Starting with this angle, several studies investigated the estimation of bone strength and stiffness *in vitro* by FEA based on different voltage and reconstruction kernels (21, 22). Later, Michalski further compared the *in vivo* femur strength estimated by FEA with different imaging reconstruction kernels (23). However, the accuracy of FEA with different reconstruction kernels on osteoporosis stratified risk assessment *in vivo* had not been fully investigated yet, which is crucial to the application of FEA in opportunistic osteoporosis screening.

Therefore, the purpose of this study was to compare the difference in vertebral mechanical properties obtained by FEA with different reconstruction kernels and evaluate the accuracy of the different reconstructed kernels in the screening and classification of osteoporosis with FEA.

## 2 Materials and methods

This retrospective study was reviewed and approved by the local institutional review board. Due to its retrospective nature, the ethics committee waived the requirement of written informed consent for participation.

### 2.1 Participants

Three sex–age-matched groups were retrospectively screened from the QCT database of our hospital, which were normal bone mass, osteopenia, and osteoporosis. All subjects underwent thoracolumbar or lumbar imaging and were identified with available raw data in the scanner’s local storage at our institution. Exclusion criteria were spinal infectious lesions, metastases, and hematological or metabolic bone disorders aside from osteoporosis.

### 2.2 Imaging

All images were acquired using the same CT scanner (128-row Somatom Definition Flash, Siemens Healthineers, Ellingen, Germany). The scanning protocol was tailored to low back pain and acquired with parameters of 120 kVp of tube voltage, 250 mAs of tube current, 128 × 0.6 mm of collimation, a pitch of 0.6, a field of view (FOV) of 199 mm, and a reconstruction thickness of 1 mm. All image raw data were reconstructed with two different statistical iterative reconstruction kernels, a smooth standard reconstruction kernel (B40f) and a sharpening bone reconstruction kernel (B70f), respectively, which are commonly used in clinical settings. A density-calibrated phantom (Mindways Inc., Austin, TX, USA) was placed in the field of view to convert HU to equivalent K_2_HPO_4_ density (*ρ*_K_2_ HPO_4__), assumed to be equal to bone ash density (*ρ*_ash_). Due to the influence of the imaging reconstruction kernel, two linear calibrating regression equations for B40f (Eq. 1) and B70f (Eq. 2) images were fit (24).


(1) $$\rho_{\text{ash}}=\rho_{{\text{K}}_{2}{\text{HPO}}_{4}}=\;-4.2\times 10^{-3}+7.0\times 10^{-4}\cdot \text{HU}$$

(2) $$\rho_{\text{ash}}=\rho_{{\text{K}}_{2}{\text{HPO}}_{4}}=\;-3.7\times 10^{-3}+7.3\times 10^{-4}\cdot \text{HU}$$

### 2.3 Finite element analysis

**Figure 1:**
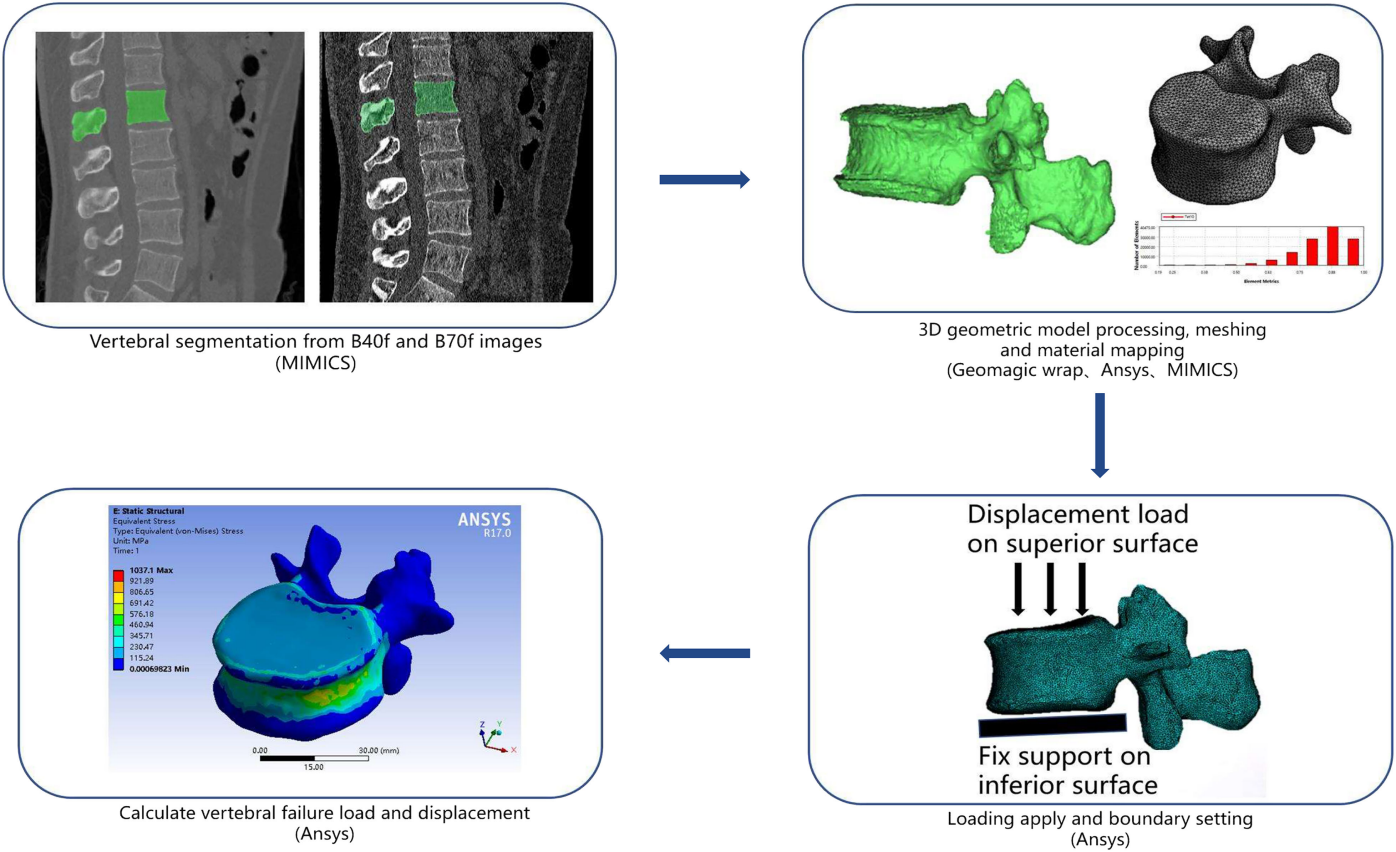
Schematic representation of geometry extraction, modeling, and analysis methodology in the current study.


Finite element analysis was performed on segmented L1 vertebra based on the MDCT datasets of B40f and B70f, respectively. If L1 was not suitable for analysis, then L2 or T12 were alternative choices in practice. The CT scan data were imported into the commercial three-dimensional (3D) medical image processing software Mimics (Materialise NV, Harrislee, Germany) for segmenting and generating 3D vertebral model. These 3D models were then imported to Geomagic software (Raindrop Company, Marble Hill, USA) for smooth geometry meshing with smooth geometry meshing with quadratic tetrahedral elements of 2-mm element edge length for downstream analysis (**Figure 1**). In consideration of bone’s nonhomogeneity, each element was assigned elastic material properties based on empirical material-mapping relations proposed by Morgan et al. (Eq. 3) (25), assuming *ρ*_ash_ is measured in grams per cubic centimeter and a ratio between *ρ*_ash_ and an apparent density (*ρ*_app_) = 0.6. Poisson’s ratio was set to 0.3 for all elements. The meshed and material-mapped 3D vertebra models were then imported into the commercial analysis software ANSYS (ANSYS Company, Canonsburg, PA, USA) for downstream FEA (25).

(3) $$E\;=4,730\times \rho_{\text{app}}^{1.56}$$

Referring to load and boundary settings in previous studies, the inferior surface of the vertebral body was fully constrained in all directions, and a displacement load was applied on the superior surface. Vertebral strength (N) was estimated using effective stress at 2% deformation, and vertebral stiffness was defined as the slope of the force-displacement curve (8).

### 2.4 Quantification of vBMD

For opportunistic screening purposes, Mindways QCT Pro Version 5.0 (Mindways Software Inc., Austin, TX, USA) with an asynchronous calibration module allowing BMD measurement from CT images without a calibration phantom had been installed in our institution (26). A new Model 4 asynchronous calibration phantom (Mindways Sofware Inc., Austin, TX, USA) was scanned for quality assurance and calibration with the same subjects’ imaging protocol weekly to maintain scanner stability. Images of subjects were sent to Mindways QCT Pro Version 5.0 to measure trabecular vBMD (mg/cm^3^) of the same vertebra that FEA was performed on.

### 2.5 Statistical analysis

All statistical analyses were performed on the software SPSS version 26.0 (IBM, Armonk, New York). Differences between FEA-B40f and FEA-B70f were compared using paired *t*-tests, and mean differences with 95% confidence intervals were computed. Linear regression analyses were used to determine the coefficient of association (*r* ^2^) between FEA results and the trabecula vBMD of the same vertebra. Spearman’s correlation test was used to analyze the correlation between FEA based on two different reconstruction kernels and the clinical classification of osteoporosis fracture risk. A ROC curve was used to compare the diagnostic efficacy of FEA based on prevalent vertebral fractures to illustrate the validity of two reconstruction kernels in fracture risk assessment.

## 3 Results

### 3.1 General characteristics of participants

Finally, 11 patients of the osteoporosis group (age: 71.1 ± 9.3, M/F patients: 3/8), every 10 patients of the osteopenia group (63.8 ± 8.2; M/F patients: 4/6) and the normal bone mass group (64.2 ± 7.8; M/F patients: 3/7), for a total of 31 subjects, were included in this study. There was no difference in gender and age among the three groups (*p* = 0.204). Of 31 subjects, 15 patients underwent lumbar CT for lumber disc herniation, seven for low back pain, four for lumbar spondylolisthesis, three for vertebral fracture, and two for spinal stenosis. For two participants in the osteoporosis group, T12 vertebral body was analyzed due to compression changes in the lumber vertebra; for one in osteoporosis and two in the osteopenia group, L2 was analyzed due to obvious osteophytosis in L1.

### 3.2 The variance of FEA measurements between two kernels

**Figure 2:**
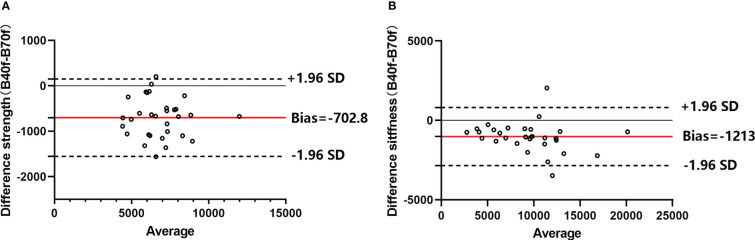
Bland–Altman plots of FE-estimated strength **(A)** and stiffness **(B)** between smooth standard kernel (B40f) and sharp bone kernel (B70f). Horizontal red lines represent the average difference between the two kernels (B40f–B70f), and the dashed line means a 95% confidence interval.

Patient-specific FEA results illustrated significant differences in the vertebral estimated strength and stiffness between B40f and B70f images (strength-B40f vs. strength-B70f: 6,457.5 ± 1,579.3 N vs. 7,482.8 ± 1,612.3 N; stiffness-B40f vs. stiffness-B70f: 8,834.3 ± 3,747.4 N/mm vs. 1,0047.4 ± 4,063.3 N/mm). Both vertebral estimated strength and stiffness were higher in FEA-based B70f (**Figures 2A, B** **)**. We further compared the differences in FEA measurements between the two kernels in three different subgroups, and the bias was similar within subgroups (**Table 1**).

**Table 1:** Estimated vertebral strength and stiffness by FEA based on standard and bone reconstruction kernels.

Kernel	Osteoporosis		Osteopenia		Normal bone mass		All subjects	
	B40f	B70f	B40f	B70f	B40f	B70f	B40f	B70f
Strength (N)	5,602.0	6,120.8	6,531.6	7,308.6	7,324.3	8,155.4	6,457.5	7,482.8
Mean absolute difference	−518.7		−777.0		−831.0		−702.8	
Mean percent difference	−9.7		−11.7		−11.1		−10.7	
*p*-value	0.003		0.000		0.000		0.000	
Stiffness (N\mm)	5,561.0	6,356.6	9,720.2	10,715.2	11,549.1	12,839.7	8,834.3	9,853.9
Mean absolute difference	−704.6		−994.9		−1,290.5		−1,213	
Mean percent difference	−14.0		−9.7		−11.2		−12.0	
*p*-value	0.000		0.001		0.024		0.000	

Absolute values are presented as average, absolute, and percent mean differences, and p-values were calculated from paired t-tests.

### 3.3 Applicability of two kernels in osteoporosis opportunistic screening

**Figure 3:**
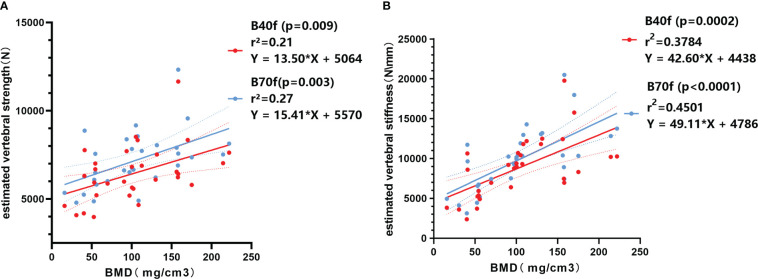
Linear regression correlation plot between BMD and FE-estimated strength **(A)** and stiffness **(B)** based on smooth standard kernel (B40f) and sharp bone kernel (B70f).

**Figure 4:**
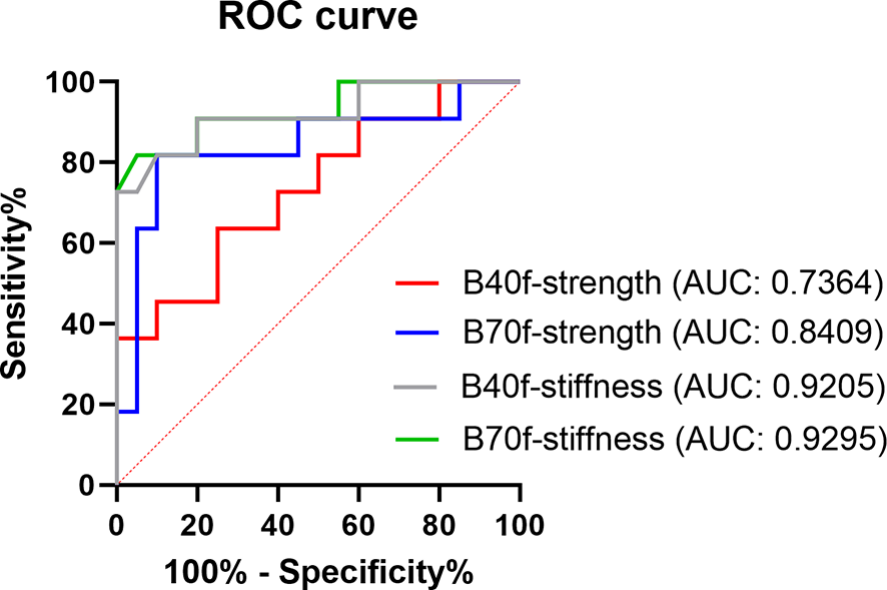
ROC curve showing the efficacy in distinguishing osteoporosis from the normal bone mass of FE-estimated strength and stiffness based on smooth standard kernel (B40f) and sharp bone kernel (B70f) images.

Subsequently, we analyzed the correlation between FEA-B40f and FEA-B70F with trabecular vBMD. The results showed that both FEA-B40f and FEA-B70f had a certain correlation with vBMD, and the correlation coefficient between FEA-B70F and vBMD was slightly higher than that of FEA-B40F in both vertebral strength and stiffness (**Figures 3A, B** **)**. Compared with vertebral strength, vertebral stiffness had a significantly higher correlation coefficient. Likewise, the ROC curve showed that stiffness-B70f was the most accurate in distinguishing osteoporosis, osteopenia, and the normal group (**Figure 4**), but FEA-B40f and FEA-B70f reveal little difference in the classification of osteoporosis. Moreover, we obtained variable cutoff values for clinical interventional thresholds of osteoporosis and osteopenia with different FEA measurements (**Table 2**).

**Table 2:** Cutoff value for osteoporosis and osteopenia by FEA based on smooth standard kernel (B40f) and sharp bone kernel (B70f) images.

	Osteoporosis	Osteopenia
Strength-B40f (N)	4,633	6,038
Strength-B70f (N)	6,498	7,129
Stiffness-B40f (N\mm)	8,676	12,314
Stiffness-B70f (N\mm)	7,478	13,016

## 4 Discussion

This study explored the impact of two reconstruction kernels commonly used in clinical musculoskeletal CT imaging—the bone-sharpening kernel and the standard kernel on vertebral estimated strength and stiffness by FEA. The reconstruction kernel is the type of filtering applied to the CT raw data to reconstruct clinical visual images (19, 20), which can significantly alter the underlying grayscale data. The sharp reconstruction kernel has the advantage of better identification of bone structures and distinction of cortical and trabecular bone at expense of high image noise. While the smooth kernel improves image density resolution and reduces image noise, it makes harder to segment bone geometry and map bone inhomogeneity (21). Our results of vertebral FEA *in vivo* agreed with those of previous studies (21–23). The estimated vertebral strength and stiffness obtained by FEA-B70f was higher than that obtained by FEA-B40f, and the consistency bias between two kernels was noted within three subgroups, indicating that it is crucial to determine the appropriate image reconstruction kernel for clinical use of CT-FEA.

We adopted diagnostic categories based on spine QCT-trabecular vBMD measurements as the gold standard since previous FEA studies verified the equivalence of FEA and vBMD osteoporotic fracture predictivity, which is superior to DXA (8, 9). Compared to QCT, the FE method takes bone geometry and the contribution of cortical bone to bone strength into consideration and requires no additional imaging hardware and radiation exposure (7). It has a promising future in opportunistic screening for osteoporosis and postoperative implant evaluation (18). With the improvement of the simulation technique, we believe the FE method will become the most valuable indicator in the screening of osteoporosis. Most of the previous studies were *in vitro* and contained a too-narrow range of bone mineral densities to reflect the impact of the reconstruction kernel on osteoporosis screening and fracture risk estimation. Therefore, the subjects we enrolled uniformly covered the range from osteoporosis to normal in order to evaluate the accuracy of FEA classification and screening for osteoporosis. Our results showed that both FEA-B40f and FEA-B70f had certain correlation with vBMD and clinical classification, but the *R* ^2^ with vBMD and accuracy of classification screening for osteoporosis by FEA-B70f was slightly higher than that by FEA-B40f, which indicated that FEA of the standard kernel can achieve similar performance in osteoporosis screening with the FEA of the bone kernel. Given that the standard kernel is widely used in clinical imaging, we recommend using FEA based on standard kernel images for the purpose of opportunistic screening.

Another strength of this study is that we established interventional thresholds for vertebral strength and stiffness based on vBMD‐defined osteoporosis, which allows FEA to identify individuals at high risk of fracture. The ROC curve was used to obtain the cutoff values of estimated vertebral strength and stiffness under the two reconstruction kernels. Although we did not adjust for gender, the strength-B40f results were closer to the intervention threshold published by Kopperdahl et al. (15), who used soft B30 kernel for FEA, while distinct from strength-B70, indicating reconstruction algorithm kernel with different frequency filter may cause different degrees of variation in estimated strength. The big variance of the cutoff values for classification of osteoporosis brought by the reconstruction kernel in our results (**Table 2**) indicated a great challenge for cross-sectional analysis of fracture risk in osteoporosis presents, where interventional thresholds of FEA estimated bone strength is an important practice guideline for clinical interpretation of fragility fractures. In order to increase acceptability and standardize the continuous methodology of FEA, further improvements are needed to increase the robustness of consolidating the proposed interventional thresholds in the setting of different imaging protocols.

Regardless of B40f or B70f images, our FE measurements showed that estimated stiffness is more suitable for the opportunistic screening of osteoporosis than estimated strength because the linear finite element model in this study makes it impossible to obtain the peak value of the stress displacement curve, which was considered a reasonable definition of FE-estimated strength. Therefore, the equivalent stress at 2% deformation is selected as the bone failure strength in this study, which has been verified in previous studies and makes the estimated stiffness more relevant with vBMD and classification of bone mass than the estimated strength because bone stiffness obtained by a linear FEA model often correlates rather well with experiment strength, while nonlinear FE analyses deliver better results for estimated strength (8).

A consensus has not been reached on which material properties are best for the prediction of osteoporotic fracture, and several “optimal” FE modeling process exist in the literature (27). Previous studies have found that different reconstruction kernels only moderately affect the pixel intensity of a water-filled phantom (22), which is also illustrated by the almost identical parameters of the calibration equations (Eqs. 1 and 2) for two kernels in this study. The power-law relationship (Eq. 3) we choose for density elasticity allows a drastic change of elasticity moduli with a small change in the image gray value. Regardless of the chosen material property, our results showed that different reconstruction kernels generated different FEA outcomes as expected, but to what extent may depend on elastic-density relationships and other modeling methods used. Apart from this, other FE modeling approaches (e.g., nonlinear FE, various loading settings, and failure criteria) will also affect osteoporosis screening or fracture prediction. Further evaluation of the impact of imaging protocols on these various FE modeling remains to be done.

This study has several limitations. Firstly, the sample size of this study is relatively small, and we did not perform a power analysis. However, the participants cover the range from osteoporotic to normal, and we believe that our results also illustrate the influence of reconstruction kernel on the use of the FEA classification to screen for osteoporosis. Nonetheless, this study was only a preliminary step; future studies with larger sample populations will help better understand any differences caused by image acquisition and FE modeling. Secondly, nonlinear FEA normally delivers better results for strength; however, the reconstruction kernel caused the nonlinear yielding behavior of the element to change and influence the relationship between models and validation outcome. Hence, we built linear FEA models. Thirdly, our study compared only two kernels in clinical scenarios; however, a smoother kernel for soft tissue imaging like B30f or B25f might be used in the most clinical practice setting, and thus future studies with a wider range of reconstruction kernels will help to better understand their influence on FEA in the clinical setting. Finally, only one scanner was used in this study, but there will be a wide variety of scanner manufacturers, and image reconstruction algorithm kernels as well as scanning protocols will vary within and between institutions, potentially leading to widely different estimates.

## 5 Conclusion

Our results revealed that the FE-estimated bone strength and bone stiffness obtained by the two reconstruction kernels reveal a significant discrepancy. FEA based on two kernels both seemed to apply to the opportunistic screening of osteoporosis, but different fragility fracture strength thresholds were noted, which has implications for the clinical management of fragility fracture. We recommend the standard reconstruction kernel for FEA because it is the most used imaging kernel and suitable for opportunistic screening of osteoporosis with considerable accuracy to differentiate osteoporosis from normal individuals. However, whether strength or stiffness is more suitable for opportunistic screening of osteoporosis by FEA may depend on the chosen modeling approach.

## Data availability statement

The raw data supporting the conclusions of this article will be made available by the authors, without undue reservation.

## Ethics statement

The studies involving human participants were reviewed and approved by Ethics Committee of Peking University Third Hospital, Beijing, China. Written informed consent for participation was not required for this study in accordance with the national legislation and the institutional requirements.

## Author contributions

HY led and coordinated this study. DJ, MN, and YZ performed the material preparation and data collection. CJ performed data interpretation and statistical analyses under the supervision of HSY. CJ wrote the majority of the manuscript. All authors read and approved the final manuscript.
